# Amyloid properties of the yeast cell wall protein Toh1 and its interaction with prion proteins Rnq1 and Sup35

**DOI:** 10.1080/19336896.2018.1558763

**Published:** 2018-12-27

**Authors:** A.V. Sergeeva, J.V. Sopova, T.A. Belashova, V.A. Siniukova, A.V. Chirinskaite, A.P. Galkin, S.P. Zadorsky

**Affiliations:** a Department of Genetics and Biotechnology, St. Petersburg State University, St. Petersburg, Russian Federation; b Vavilov Institute of General Genetics, St. Petersburg Branch, Russian Academy of Sciences, St. Petersburg, Russian Federation

**Keywords:** Functional amyloids, prions, yeast, cell wall

## Abstract

Amyloids are non-branching fibrils that are composed of stacked monomers stabilized by intermolecular β-sheets. Some amyloids are associated with incurable diseases, whereas others, functional amyloids, regulate different vital processes. The prevalence and significance of functional amyloids in wildlife are still poorly understood. In recent years, by applying new approach of large-scale proteome screening, a number of novel candidate amyloids were identified in the yeast *Saccharomyces cerevisiae*, many of which are localized in the yeast cell wall. In this work, we showed that one of these proteins, Toh1, possess amyloid properties. The Toh1-YFP hybrid protein forms detergent-resistant aggregates in the yeast cells while being expressed under its own *P_TOH1_* or inducible *P_CUP1_* promoter. Using bacterial system for generation of extracellular amyloid aggregates C-DAG, we demonstrated that the N-terminal Toh1 fragment, containing amyloidogenic regions predicted *in silico*, binds Congo Red dye, manifests ‘apple-green’ birefringence when examined between crossed polarizers, and forms amyloid-like fibrillar aggregates visualized by TEM. We have established that the Toh1(20–365)-YFP hybrid protein fluorescent aggregates are co-localized with a high frequency with Rnq1C-CFP and Sup35NM-CFP aggregates in the yeast cells containing [*PIN^+^*] and [*PSI^+^*] prions, and physical interaction of these aggregated proteins was confirmed by FRET. This is one of a few known cases of physical interaction of non-Q/N-rich amyloid-like protein and Q/N-rich amyloids, suggesting that interaction of different amyloid proteins may be determined not only by similarity of their primary structures but also by similarity of their secondary structures and of conformational folds.

## Introduction

Amyloids are protein fibrils that are composed of stacked monomers stabilized by intermolecular β-sheets. Universal characteristics of amyloids include high resistance to ionic detergents, such as sodium dodecyl sulfate (SDS), and proteases [1–3], and propensity to bind amyloid-specific dyes thioflavines T and S [4], and Congo red [5]. Binding of amyloids with Congo red is accompanied with an apple-green birefringence in polarized light [5]. Along with pathological amyloids connected with different amyloid-associated disorders, there are functional amyloids that perform various vital functions and have been found in a broad range of living organisms [6–9]. Infectious amyloids, called prions, cause incurable neurodegenerative diseases in human and mammals [10] and heritable phenotypical traits in unicellular eucaryota [11,12]. Among the most studied prions of lower eukaryotes there are cytoplasmic determinants of the yeast *Saccharomyces cerevisiae* – [*PSI^+^*] [13], the prion form of the translation termination factor eRF3 (Sup35) [11,14], and [*PIN^+^*] [15], the prion form of the Rnq1 protein [16,17].

One of the characteristic features of amyloids is their ability to interact with heterologous amyloid proteins. Interactions of amyloid proteins may be mediated by the similarity of their primary structures. For yeast prions formed by glutamine/asparagine rich (Q/N-rich) proteins maintenance or promotion/inhibition of the *de novo* appearance of one prion in the presence of another have been shown [16,18–21]. Moreover, Q/N rich prions may promote the seeding of non-inheritable Q/N-rich amyloids as well, such as human amyloid form of huntingtin [22,23]. However, in recent years the cases of interactions between amyloid proteins with dissimilar primary structures, in particular between Q/N-rich and non Q/N-rich amyloid proteins, were reported. For instance, Q/N-rich prions [*PIN^+^*] and [*PSI^+^*] promote aggregation of non-Q/N-rich fungal prion protein Het-s and of human amyloid protein transthyretin, respectively [24,25]. These data may suggest that not only primary structures but also conformational folds of amyloid proteins determine their ability to interact.

Recently developed methods of large-scale proteome screening for amyloid-forming proteins [26–28] provided a significant progress in discovery of novel amyloids. Screening of the *S. cerevisiae* proteome using the PSIA-LC-MALDI method [28] revealed a number of potential constitutive amyloids, significant part of which was represented with the cell wall proteins. Two of these proteins, Gas1 and Ygp1, displayed amyloid properties in yeast cells and in the bacteria-based С-DAG system [29].

Yeast cell wall prevents cell lysis and protects a cell from harmful environmental conditions. It represent a complex multilayer structure consisting of a fibrillar network, formed by glucanes and chitine, to which mannoproteins are attached [30]. In different yeast species, a capacity to form functional amyloid fibrils was shown earlier for several cell wall proteins including adhesins Als3 and Als5 in *Candida albicans* and Flo1 and Muc1 in *S. cerevisiae* [31,32], and Bgl2 protein fibrils that stabilize the cell wall of *S. cerevisiae* [33,34]. It is presumed that the yeast cell wall may contain an ensemble of proteins in amyloid state participating in the maintenance of its structure [29,33].

In this work we studied amyloid properties of the yeast cell wall protein, Toh1, which was revealed in the fraction of SDS-resistant aggregates by PSIA-LC-MALDI [29]. Toh1 is a GPI-anchored cell wall protein with unknown function [35]. We show that the Toh1 protein demonstrates amyloid properties in yeast cells under native conditions and in the bacterial C-DAG system. These data implies that Toh1 can be another potential amyloid that functions in the cell wall of *S. cerevisiae*, and support the statement that a complex of interacting amyloid-forming proteins may be an important constituent of the yeast cell wall determining its structure, plasticity and stability. We demonstrate that Toh1 fragment fused to YFP interacts physically with the amyloid aggregates of the prion proteins Rnq1 and Sup35 in the yeast cells, despite the fact that Toh1 is non Q/N-rich protein which is dissimilar in primary structure with Q/N-rich Rnq1 and Sup35 proteins. This physical interaction between non Q/N-rich and Q/N-rich proteins speaks in favor of the idea that interactions of different amyloid proteins are determined by their structural and conformational similarity rather than by simple similarity of amino acid sequences.

## Materials and methods

### Yeast and bacterial strains

The *S. cerevisiae* strain BY4742 (*MATα his3Δ1* leu*2Δ0* lys*2Δ0 ura3Δ0*) (Invitrogene) and 74-D694(*MATa ade1-14 his3-Δ200 ura3-52 leu2-3,112 trp1-289*) [36] were used in this work. To test the amyloid properties of the Toh1 protein fragment in the bacterial expression system C-DAG (Curli-Dependent Amyloid Generator), we used the *Escherichia coli* strain VS39 (*F ^–^*, [*araD139*]*B/r, Δ(argF-lac)169, λ−, e14−, flhD5301, Δ(fruK-yeiR)725 (fruA25), relA1, rpsL150* (*strR), rbsR22, Δ(fimBfimE)632(::IS1), deoC1, Δ(csgBAC) (::kanR)*) kindly provided by A. Hochschild. This strain is a derivative of the strain MC4100 carrying deletions of the curli-encoding genes, *csgA, csgB*, and *csgC*, generated by gene replacement with the *neo* gene providing resistance to kanamycin. VS39 contains a pACYC-derived pVS76 plasmid that directs the synthesis of the outer-membrane curli protein, CsgG, under the control of the IPTG-inducible *lacUV5* promoter and carries the *cat* gene providing resistance to chloramphenicol [37].

### Plasmids

The plasmid YGPM16d14 from the yeast genomic tiling library YSC4613 (Open Biosystems, USA) containing *TOH1* gene was used for PCR amplification of this gene and of its fragments. The plasmid pRS415-CUP1-YFP was used as a vector for obtaining plasmids with the hybrid *TOH1-YFP* gene under *P_TOH1_* and *P_CUP1_* promoters. This plasmid was obtained by cloning ApaI-SacI fragment of pU-CUP1-YFP plasmid [38], containing *P_CUP1_* promoter and *YFP* coding sequence, into pRS415 vector.

The plasmid pTOH1-YFP contains the chimeric *TOH1-YFP* gene under the *P_TOH1_* promoter. To construct this plasmid, PCR-generated *P_TOH1_-TOH1* fragment, amplified using the primers ForPromTOH1 and RevTOH1 (Table 1), was digested with SalI and BamHI and inserted into the SalI–BamHI digested pRS415-CUP1-YFP plasmid. The plasmid pRS415-CUP-TOH1-YFP contains chimeric gene, coding for full size Toh1 fused to YFP, under the *P_CUP1_* promoter. To construct this plasmid, PCR-generated *TOH1* coding sequence, amplified using the primers ForTOH1 and RevTOH1 (Table 1), was digested with PstI and BamHI and inserted into the PstI–BamHI digested pRS415-CUP1-YFP plasmid. The pRS415-CUP-Toh1(20–365)-YFP plasmid carries chimeric gene, coding for Toh1 fragment (amino acids 20–365) fused to YFP, under the *P_CUP1_* promoter. To construct this plasmid, PCR-generated fragment of *TOH1* amplified using the pair of the primers ForTOH1(20) and RevTOH1(365) (Table 1), was digested with PstI and BamHI and inserted into the PstI–BamHI digested pRS415-CUP1-YFP plasmid. The plasmid pCUP-GFP [39] carrying GFP coding sequence under *P_CUP1_* promoter was used as a negative control for aggregation. pCUP-RNQ1-CFP [23] is pRS316 based plasmid encoding Rnq1 prion domain (amino acids 153–405) fused to CFP. pCUP-NM-CFP is pRS313 based plasmid obtained from pNM-YFP [23] by the replacement of YFP sequence with CFP from pCUP-RNQ1-CFP plasmid.

**Table 1.** Primers used in this study.

Primer	Sequence 5ʹ to 3’
ForPromTOH1	CTAAGTCGACACACCTTGAAAAAATATCGTTAC
ForTOH1	CCAGCTGCAGATGTTACAGAGTATAGTCCTT
RevTOH1	GTTTGGATCCAATCAAAACCACTTGCGTAAAG
ForTOH1(20)	TATCTGCAGATGGCACCTCAGTCGTACC
RevTOH1(365)	TATGGATCCCGAGGTGGTCGAAGGC
TOH1F(1)	CTTGCGGCCGCAATGTTACAGAGTATAGTCCTTTC
TOH1R(163)	CCGTCTAGATTAAATCAAAGTGCCATCTG
TOH1F(136)	CTTGCGGCCGCATTAGGAAATGCTTTGTCCT
TOH1R(321)	CGCTCTAGATTATATGAAAGAAACGACATTTCCA

The plasmids pExport (pVS72) and pVS105 [40] were kindly provided by A. Hochshild. These plasmids contain chimeric genes encoding the signal sequence of CsgA protein fused to the M-domain (pVS105) or NM-domain (pVS72) of the Sup35 protein of *S. cerevisiae*, under the *BAD* promoter induced by arabinose. The plasmid pVS72 was used as a vector for cloning selected fragment of *TOH1* gene. The plasmids pVS-TOH1(1–163) and pVS-TOH1(136–321) contain chimeric genes encoding CsgA signal sequence fused to Toh1(1–163) and Toh1(136–321) fragments, correspondingly, under the *BAD* promoter. To obtain these plasmids, the *TOH1* gene fragments generated by PCR with the pairs of primers TOH1F(1) and TOH1R(163), and TOH1F(136) and TOH1R(321), respectively, were digested with NotI and XbaI and substituted for the XbaI-NotI fragment containing the *SUP35NM* in the pVS72 plasmid.

### Protein analysis

Preparation of protein lysates was performed as described previously [41].

Fractionation of the crude cell lysate was performed at 3000 rpm (~875 g) for 5 min at 4°C, the aliquots of the resulting fractions of the cell debris and cell lysate were boiled for 10 min in the sample buffer (final concentration 25 мM Tris-HCI, pH 6,8, 5% 2-mercaptoethanol, 2% SDS, 0,05% Bromphenol blue, 10% glycerin) and analyzed by Western blotting.

Semi-denaturing detergent agarose gel electrophoresis (SDD-AGE)[42,43] was performed using 1% agarose gel. Before loading onto a gel, protein extracts were either treated for 10 min with 1% SDS at room temperature or boiled for 10 min with 2,5% SDS. Then, the extracts were subjected to SDD-AGE and transferred onto Immobilon-P PVDF membrane (GE Healthcare, USA). Proteins fused with YFP were reacted with polyclonal chicken primary antibodies against GFP (ab13970) (Abcam, Great Britain). Reactions with the secondary antibodies and chemiluminescent detection were performed using the Amersham ECL Prime Western Blotting Detection Reagent kit (GE Healthcare, USA).

### Fluorescent microscopy and FRET analysis

Fluorescent assay for YFP fusion proteins was performed with Leica DM6000B microscope (Leica Microsystems GmBH, Germany) and ‘Leica QWin standart V. 3.2.0.’ software. The fluorescence was analyzed using YFP cube (Leica Microsystems GmBH, Germany) equipped with 535-nm barrier and 500-nm excitation filters. For quantification of fluorescent aggregates formation cells were grown for 72 h in synthetic selective media, containing 100 mM CuSO_4_ when the proteins under study were expressed from *P_CUP1_* promoter.

Co-localization of Toh1(20–365)-YFP aggregates with Sup35NM-CFP and Rnq1C-CFP aggregates was evaluated visually and quantitatively. Cells from independent co-transformants producing Toh1(20–365)-YFP together with Sup35NM-CFP or Rnq1C-CFP after 72 h induction in selective media containing 100 mM CuSO_4_ were analyzed under confocal microscope Leica TCS SP5 for aggregates, and images were captured separately for both YFP and CFP channels. To excite CFP-containing structures, an argon laser with a wavelength of 458 nm was used, the signal was detected within the range 461–510 nm. To detect YFP-containing chimeric protein structures, an argon laser with a wavelength of 514 nm was used, the signal was detected in the range of 518–580 nm. Co-localization was analyzed by merging the images from two channels using LAS AF application Wizard. The frequencies of co-localization were determined using the Fiji software as ratios of the number of cells with co-localized Toh1(20–365)-YFP/Sup35NM-CFP or Toh1-(20–365)-YFP/Rnq1C-CFP aggregates to the number of cells producing both Toh1(20–365)-YFP and Sup35NM-CFP or Rnq1C-CFP fluorescent proteins.

To investigate the possibility of physical interaction between aggregated proteins, the FRET AP method (Fluorescence Resonance Energy Transfer Acceptor Photobleaching) was used on cells with co-localized Toh1-YFP/Sup35NM-CFP and Toh1-YFP/Rnq1-CFP aggregates. The FRET efficiency was calculated automatically using the LAS AF software.

### Analysis of amyloid fibril formation in the bacteria-based system C-DAG

All the tests to verify the amyloid properties of different fragments of the Toh1 protein using bacterial expression system C-DAG (curli-dependent amyloid generator) were performed as described earlier [37,40]. *E. coli* strain VS39 was transformed with the plasmid carrying the gene encoding tested Toh1 fragment fused to CsgA signal sequence. VS39 transformants with pVS72 and pVS105 encoding the CsgA_SS_-Sup35NM and CsgA_SS_-Sup35M proteins were used as positive and negative controls for amyloid generation, respectively. To perform the tests for colony color phenotype and CR birefringence, spots of the transformants were grown for 5 days at 22° C on the inducing medium with Congo Red (LB supplemented with 100 mg/l ampicillin, 25 mg/l chloramphenicol, 0.2% w/v L-arabinose, 1 mM IPTG and 10 mg/l Congo Red). To perform CR birefringence analysis, suspensions of the cells grown on CR-containing medium were spotted on a glass slide and analyzed between cross polarizers on the inverted microscope Leica DMI6000 B. Images were acquired using the Leica Application Suite software. To perform transmission electron microscopy analysis, spots of the transformants were grown for 5 days at 22 °C on the inducing medium without Congo Red (LB supplemented with 100 mg/l ampicillin, 25 mg/l chloramphenicol, 0.2% w/v L-arabinose and 1 mM IPTG). Cell suspensions were then analyzed on the transmission electron microscope Jeol JEM-2100 (‘JEOL Ltd’, Japan).

### Bioinformatic methods and statistical analysis

The bioinformatic algorithms ArchCandy [44,45] and Amylpred2 [46] were used for determining potentially amyloidogenic regions in the amino acid sequence of the Toh1 protein.

Statistical analysis was performed using Microsoft Excel software for Mac OS, version 16.16.2. Data was checked for normality and two-tailed t-test was applied to determine the statistical significance and p-value less than 0.05 was considered to be significant.

## Results

### Toh1-YFP protein forms fluorescent aggregates in *S.cerevisiae* cells

**Figure 1.**
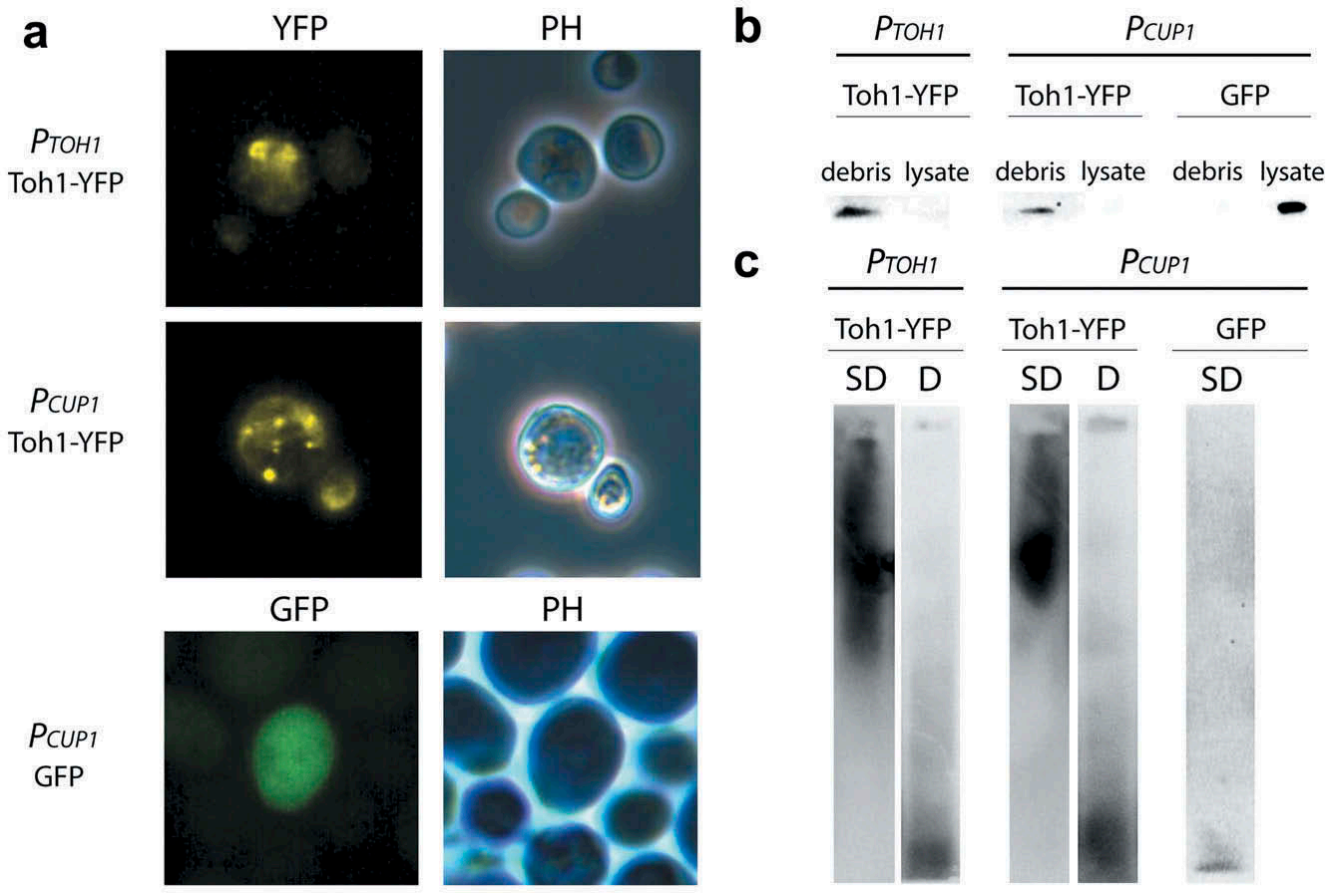
Toh1-YFP protein forms detergent-resistant aggregates in yeast cells that are revealed predominantly in the cell debris. (a) Localization of the Toh1-YFP aggregates in yeast cells. Transformants of BY4742 strain expressing TOH1-YFP and GFP genes were grown on -Leu selective media for 48 h prior to fluorescence microscopy. In the case of the constructs under *P_CUP1_* promoter media was supplemented with 100 μM CuSO_4_. (b) Fractionation of the crude cell lysates extracted from BY4742 cells expressing the Toh1-YFP protein showing predominating localization of Toh1-YFP in the debris fraction. Cells expressing Toh1-YFP protein were harvested after 48h, lysed and the crude cell lysate was separated into debris and lysate fractions followed by the denaturating treatment of the samples (2,5% SDS, 95°C). Samples were resolved in 10% SDS-PAGE and immunoblotted using anti-GFP antibody (ab32146 (Abcam, Great Britain)). (c) SDD-AGE of the crude protein lysates extracted from BY4742 cells expressing Toh1-YFP protein showing the capacity of Toh1-YFP to form SDS-resistant aggregates. The debris fractions of the crude protein lysates were incubated under semi-denaturing (SD, 1% SDS at room temperature) and denaturing (D, 2,5% SDS, 95°C) conditions. Samples were resolved on 1,5% SDS-AGE and immunoblotted using anti-GFP antibody (ab32146 (Abcam, Great Britain)). Cells expressing GFP were used as a negative control for aggregation.

To determine whether the Toh1 protein has amyloid-like properties we primarily estimated the ability of the Toh1 fused with YFP to aggregate in the *S. cerevisiae* cells. Transformants of the yeast strain BY4742 with the plasmids pRS415-TOH1-YFP and pRS415-CUP-TOH1-YFP, expressing the *TOH1-YFP* gene under authentic *P_TOH1_* and inducible *P_CUP1_* promoters, respectively, were grown in selective medium omitting leucine, and transformants with control plasmid pCUP-GFP, expressing GFP – in the medium omitting uracil. In the case of transformants with pRS415-CUP-TOH1-YFP and pCUP-GFP the media were supplemented with 100 mM CuSO_4_. Fluorescent microscopy of the transformants showed that Toh1-YFP forms fluorescent foci when *TOH1-YFP* gene is expressed either from *Р_TOH1_*, or from *P_CUP1_* promoter (Figure 1(a)). The cells with visible fluorescent aggregates accounted for about 7% of total cell number in the transformants carrying *P_CUP1_-TOH1-YFP*, and about 19% – in the transformants carrying *P_TOH1_-TOH1-YFP*. Cells of control transformants carrying pCUP-GFP demonstrated diffuse fluorescence (Figure 1(a)). Thus, Toh1-YFP protein aggregates in *S.cerevisiae* cells.

### Toh1-YFP protein is revealed predominantly in the cell debris in a form of detergent-resistant polymers

To estimate a proportion of the hybrid Toh1-YFP protein included in the high molecular weight aggregates we used the method of the fractionation of the cell lysate. First, crude lysates of the transformants of BY4742 strain with the plasmids pRS415-TOH1-YFP, pRS415-CUP-TOH1-YFP, and pCUP-GFP were centrifugated at 3000 rpm (~875 g) for 5 min and thus divided into fractions of the cell debris and cell lysate. Then aliquots of two fractions were analyzed by SDS-PAGE and Western blotting with antibody to GFP. In the case of the transformants carrying pRS415-CUP-TOH1-YFP and pRS415-TOH1-YFP essentially all Toh1-YFP protein was found in the fraction of the cell debris and was almost absent in the cell lysate (Figure 1(b)). In a control experiment with transformants carrying pCUP1-GFP plasmid the GFP protein was detected in the fraction of cell lysate (Figure 1(b)). These results may reflect the fact that the majority of Toh1-YFP protein is included in the aggregates of large size that sedimented completely at low-speed centrifugation, and/or that Toh1-YFP is tightly associated with cell wall and/or cell membrane, fragments of which drag essentially all Toh1-YFP into the cell debris during centrifugation.

We also analyzed Toh1-YFP protein from the cell debris of BY4742 transformants carrying pRS415-TOH1-YFP plasmid using the semi-denaturing detergent agarose gel electrophoresis (SDD-AGE) assay. Cell lysate of BY4742 strain transformed with pCUP1-GFP plasmid was used as a control. Equal amounts of debris were either incubated in the buffer with 1% SDS at room temperature, which lead to the dissolution of only SDS-sensitive complexes, or boiled in the buffer with 2% SDS, which lead to dissolution of both SDS-sensitive and SDS-resistant complexes including amyloid polymers (see ‘Materials and methods’). Then the samples were analyzed by the electrophoresis in agarose gel and Western blotting with antibody to GFP. We detected Toh1-YFP high molecular weight polymers in unboiled sample and predominantly monomeric Toh1-YFP protein in the boiled sample (Figure 1(c)). Control GFP protein was presented only in monomeric form (Figure 1(c)). Thus, Toh1-YFP forms high-weight SDS-resistant aggregates in the *S. cerevisiae* cells.

### Identification of potentially amyloidogenic regions in Toh1 protein by bioinformatic algorithms

By using bioinformatic algorithms ArchCandy and Amylpred2 we determined potentially amyloidogenic regions in the amino acid sequence of Toh1 protein. The results of the analysis are presented in the Table 2. ArchCandy algorithm has revealed 2 potentially amyloidogenic regions in Toh1, aa 136–162 and 291–317. Amylpred 2 algorithm also has revealed potentially amyloidogenic peptides but did not allow localizing any extended amyloidogenic region in the protein. Note that 4 regions of those identified with Amylpred 2 overlap significantly with 2 regions revealed with ArchCandy (Table 2).

**Table 2.** Potentially amyloidogenic regions of the Toh1 protein revealed with the bioinformatic algorithms ArchCandy and Amylpred2.

Algorithm	Parameters of analysis	Potentially amyloidogenic regions identified in Toh1 amino acid sequence
ArchCandy	Scoring Threshold = 0.575	136–162; 291–320
Amylpred2	Basic	72–93; 137–141; 157–161; 170–177; 189–211; 223–236; 291–296; 305–325; 351–359

### Analysis of the amyloid properties of Toh1 protein fragments in the bacteria-based expression system C-DAG

To further verify amyloid-like properties of the Toh1 protein, we used the bacteria-based system for generating extracellular amyloid aggregates called C-DAG (Curli-dependent amyloid generator) [37,40]. This system represents a convenient cell-based alternative to conventional methods of the study of protein’s amyloid properties *in vitro* as it does not require either purification of the studied protein or optimization of conditions for amyloid fibril assembly *in* *vitro*. C-DAG system relies on the ability of *E. coli* cells to generate surface associated amyloid fibrils (curli) composed of the CsgA and CsgB proteins. N-terminal bipartite signal sequence (CsgA_SS_), which is present in both proteins, directs them to the cell surface through the general Sec translocon and curli-specific pore-like structure in the outer membrane formed by the CsgG protein. Heterologous amyloidogenic proteins fused to CsgA_SS_ are similarly exported to the cell surface where they form amyloid fibrils. These extracellular amyloid fibrils can be easily detected *in vivo* using a colony color assay and studied by diverse methods including CR birefringence analysis and transmission electron microscopy [40].

**Figure 2.**
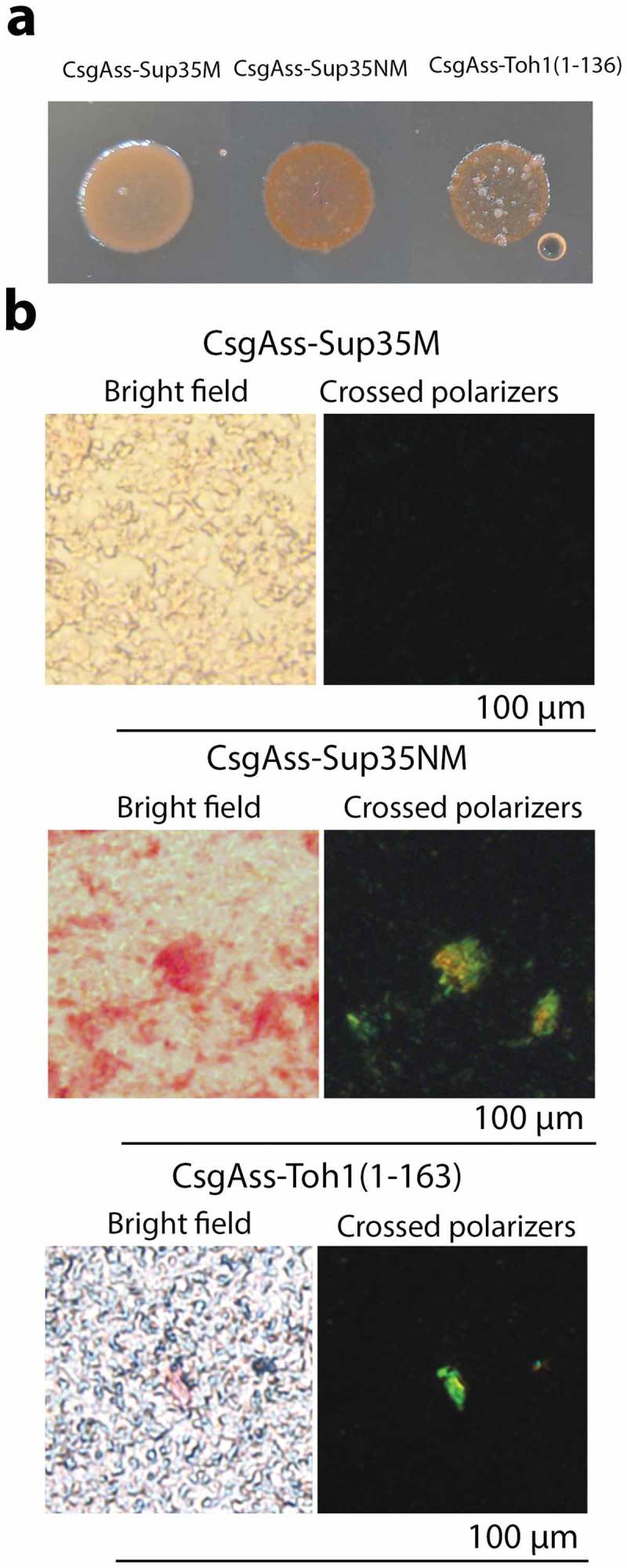
Analysis of the amyloid properties of the CsgAss-Toh1(1–163), CsgAss-Sup35NM and CsgAss-Sup35M proteins in the bacterial C-DAG system. (a) Colony color assay of the cells when plated on agar containing CR showing CR binding for colonies expressing CsgAss-Toh1(1–163) and CsgAss-Sup35NM. (b) Micrographs of CsgAss-Toh1(1–163), CsgAss-Sup35NM and CsgAss-Sup35M scraped cell samples harvested from CR-containing agar. Extracellular material binds CR (left) and displays apple-green birefringence when viewed between crossed polarizers (right). The cultures producing the CsgAss-Sup35NM and CsgAss-Sup35M proteins were taken as positive and negative controls, respectively.

**Figure 3.**
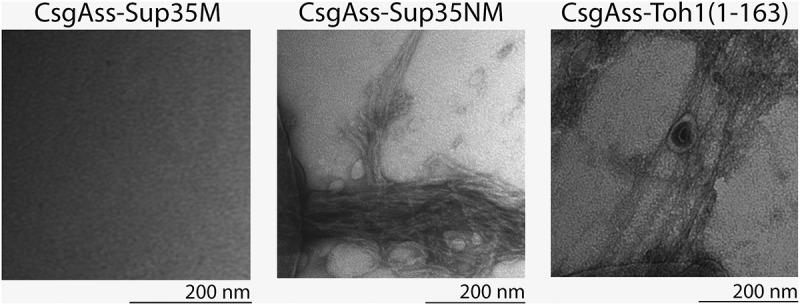
Electron micrographs of amyloid fibrils formed by secreted CsgAss-Toh1(1–163) and CsgAss-Sup35NM proteins. Scale bar, 200 nm.

We studied the amyloid-like properties of two Toh1 protein fragments (aa 1–163, aa 136–321) in the C-DAG system. The first fragment contains one amyloidogenic region predicted by ArchCandy and three regions predicted by Amylpred 2, and the second fragment – two regions predicted by ArchCandy and seven regions predicted by Amylpred 2. *E.coli* VS39 strain was transformed with the plasmids pVS-TOH1(1–163) and pVS-TOH1(136–321) encoding CsgA_SS_ fused to Toh1(1–163) and Toh1(136–321) fragments, respectively. VS39 transformants with pVS72 and pVS105 plasmids providing the expression of CsgA_SS_-Sup35NM and CsgA_SS_-Sup35M proteins, respectively, were used as positive and negative controls for amyloid aggregation. Colony color phenotype test showed that production of CsgA_SS_-Toh1(1–163) and CsgA_SS_-Toh1(136–321) chimeric proteins as well as of CsgA_SS_-Sup35NM resulted in red colonies of transformants on inducing medium containing Congo red. The transformants with the pVS105 plasmid producing CsgA_SS_-Sup35M protein were pale on this medium (Figure 2(a); Figure S1). These data suggest that the CsgA_SS_-Toh1(1–163) and CsgA_SS_-Toh1(136–321) proteins form extracellular amyloid-like aggregates binding Congo red, which results in the red color of colonies. To confirm this, we performed the test for CR birefringence by polarization microscopy and transmission electron microscopy of the transformants. We showed that the CsgA_SS_-Toh1(1–163), CsgA_SS_-Toh1(136–321) and CsgA_SS_-Sup35NM proteins, but not CsgA_SS_-Sup35M, bind Congo red and display apple-green birefringence when examined between crossed polarizers (Figures 2(b); Figure S2; Figure S4) that is discriminative characteristic of amyloid fibrils [47]. The transmission electron microscopy analysis of the cell suspension of transformants scraped from inducing medium revealed extracellular fibrils of the CsgA_SS_-Toh1(1–163), CsgA_SS_-Toh1(136–321) and CsgA_SS_-Sup35NM proteins, but not of the negative control protein CsgA_SS_-Sup35M (Figure 3; Figure S3; Figure S5).

Taken together, the data obtained show that the yeast cell wall protein Toh1 forms detergent resistant aggregates in the yeast cells and their fragments generate amyloid fibrils in the bacteria-based expression system C-DAG.

### Toh1(20–365)-YFP protein interacts with amyloid aggregates of Sup35 and Rnq1 proteins

Interactions of amyloid proteins with similar primary structure, in particular Q/N-rich amyloids, are studied in sufficient detail in the yeast model [16,18–21]. Significantly less is known about interactions of the amyloids with dissimilar primary structures, particularly interactions between Q/N-rich and non-Q/N-rich amyloids. We used the expression of the fragment of non-Q/N-rich protein Toh1 fused to YFP in the yeast strains carrying [*PIN^+^*] and [*PSI^+^*] prions formed by Q/N-rich Rnq1 and Sup35 proteins, respectively, as a model to study such interactions. In order to analyze the interaction of these proteins in the cytoplasm, we have constructed the pRS415-CUP-Toh1(20–365)-YFP plasmid encoding internal Toh1 fragment (aa 20–365) fused to YFP. The chimeric Toh1(20–365)-YFP protein lacks N-terminal signal peptide and С-terminal peptide of Toh1, which are necessary for the transport and incorporation of Toh1 into the cell wall [35], and is presumed to be localized in cytoplasm as well as Rnq1 and Sup35 proteins.

**Figure 4.**
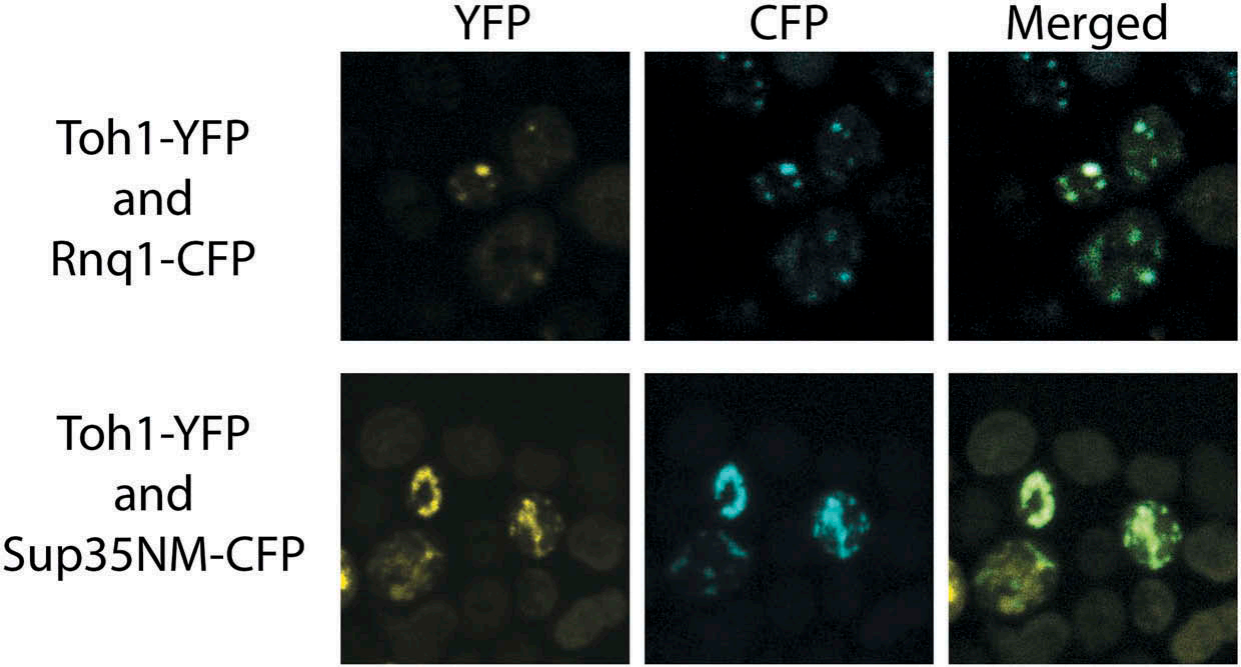
Co-localization of the fluorescent aggregates of Toh1(20–365)-YFP protein with aggregates of Sup35NM-CFP and Rnq1C-CFP proteins. Images were captured under YFP and CFP channels and merged using Fiji software.

To estimate the ability of the Toh1(20–365)-YFP protein to physically interact with the aggregates of Rnq1 and Sup35 proteins in the yeast cells, we co-transformed [*PIN^+^*] [*psi^−^*] and [*PIN^+^*] [*PSI^+^*] variants of the 74-D694 strain with the plasmid pRS415-CUP-Toh1(20–365)-YFP and one of the plasmids pCUP-RNQ1-CFP or pCUP-NM-CFP encoding prion-forming domains of Rnq1 and Sup35, respectively, fused to YFP. Using confocal fluorescent microscopy, we found that in the cells that contained visible aggregates of both Toh1(20–365)-YFP and Sup35NM-CFP, these aggregates co-localized with a frequency about 75 %, and in the cells with visible aggregates of both Toh1(20–365)-YFP and Rnq1С-CFP a frequency of co-localization amounted for about 99 % (Figure 4). Moreover, generally we did not observe an even distribution of Toh1(20–365)-YFP in the cells with Sup35NM-CFP or Rnq1C-CFP aggregates.

To confirm that the Toh1(20–365)-YFP protein physically interacts with the Sup35NM-CFP and Rnq1C-CFP proteins in the aggregates, we used the FRET method based on radiationless transmission of energy from a donor fluorophore molecule to an appropriately arranged acceptor fluorophore molecule. The effectivity of energy transfer varies inversely with the sixth power of the distance between donor and acceptor fluorophores so energy transfer occurs effectively only in the case of physical interaction between the proteins under study, and is ineffective when the proteins have close localization in the cell but do not interact physically [48]. The effectivity of the FRET in the transformants expressing Toh1(20–365)-YFP/Sup35NM-CFP and Toh1(20–365)-YFP/Rnq1С-CFP in our experiments amounted about 4–5 % and was significantly higher than in a negative control (transformants expressing YFP and Sup35NM-CFP) (Table 3). Thus, the data obtained confirmed the physical interaction of Toh1(20–365)-YFP protein with Sup35NM-CFP and Rnq1С-CFP proteins.

**Table 3.** Estimation of physical interaction of Toh1(20–365)-YFP with Sup35NM-CFP and Rnq1C-CFP proteins using the FRET method.

Strain	Proteins	FRET efficiency (%)
74-D694 [*PSI^+^*] [*PIN^+^*]	Sup35NM-YFP and Sup35NM-CFP	8.6 ± 1.4
	YFP and Sup35NM-CFP	0.5 ± 0.2
	Toh1-YFP and Sup35NM-CFP	3.9 ± 0.8
	Toh1-YFP and Rnq1C-CFP	4.8 ± 1.0
74-D694 [*psi^−^*] [*PIN^+^*]	Toh1-YFP and Rnq1C-CFP	5.4 ± 1.2

## Discussion

In this work we analyzed amyloid properties of the yeast cell wall protein Toh1 in the *S. cerevisiae* cells and in bacteria-based system C-DAG. Toh1 protein was identified earlier as a potential functional amyloid [29] using new method of large-scale proteome screening for amyloids, PSIA-LC-MALDI, which opens promising opportunities for identification of the landscapes of amyloids in different cells, tissues and organisms [28]. Toh1 was revealed with a high reliability in all studied *S. cerevisiae* strains along with a number of other cell wall proteins. Two of them, Ygp1 and Gas1, were confirmed to possess amyloid-like properties in yeast cells and in the bacterial system C-DAG [29]. In the work presented here, we showed that Toh1 also demonstrates amyloid-like properties. We have shown that the Toh1 protein, fused to YFP, forms visible fluorescent aggregates in yeast cells, both in overproduction conditions and being expressed from the native *P_TOH1_* promoter. Unlike Ygp1-YFP and Gas1-YFP chimeric proteins [29], Toh1-YFP was predominantly revealed in our experiments in the cell debris. The aggregates of the Toh1-YFP were resistant to the treatment with 1% SDS at RT, which is one of the main discriminative characteristics of amyloids. These results may suggest that in a cell the majority of the Toh1-YFP hybrid protein is represented with SDS-resistant aggregates that have very large size and completely sedimented at low-speed centrifugation, or/and are tightly associated with the cell membrane or/and with the cell wall. The last possibility is in accordance with the data that Toh1 is GPI-anchored protein localized predominantly to the cell wall [35].

To confirm amyloid-like properties of the Toh1 protein, we used bacteria-based system for generating extracellular amyloid aggregates called C-DAG [37,40]. C-DAG method relies on the natural ability of *E. coli* cells to elaborate amyloid fibrils at a cell surface and can be considered as a simple and convenient alternative to traditional methods for confirming the amyloid properties of the tested protein, such as the preparation and study of amyloid fibrils of recombinant protein *in vitro*. Moreover, as C-DAG is a cell-based system, it can be regarded as a more physiological technique of testing protein’s amyloidogenic properties comparing to *in vitro* experiments. In several studies, it was shown that the results obtained using C-DAG generally strongly resemble the results of the study of protein’s fibrils formation *in vitro* [37,40,49,50] or *in vivo* genetic tests for prion inheritance [51].

**Figure 5.**
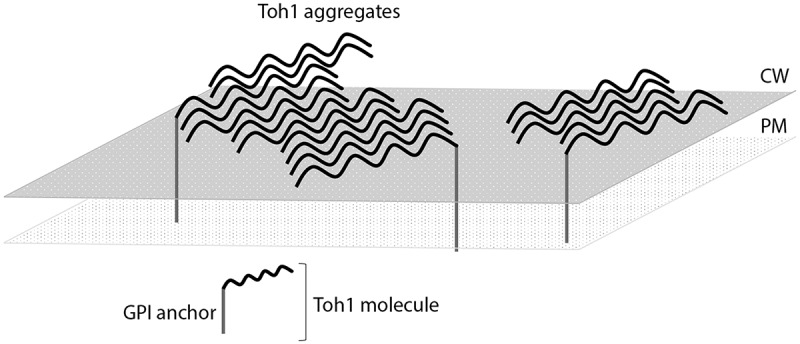
The proposed model for the organization of the Toh1 amyloid-like aggregates in the *S. cerevisiae* cell wall. One part of protein molecules is covalently linked with the cell wall glycane scaffold by means of GPI anchor, and another part of protein is linked with the GPI-anchored Toh1 molecules through hydrogen bonds, forming SDS-resistant amyloid-like aggregates.

We estimated the ability of the N-terminal Toh1 fragment (aa 1–163) and of Toh1 (aa 136–321) fragment fused to signal sequence of the CsgA protein of *E.coli* (CsgA_SS_) to form extracellular amyloid-like fibrillar aggregates in the C-DAG system. The examined Toh1 fragments contain potentially amyloidogenic regions, predicted by the ArchCandy and Amylpred2 algorithms. The chimeric proteins CsgA_SS_-Toh1(1–163) and CsgA_SS_-Toh1(136–321) were shown to form extracellular amyloid-like fibrillar aggregates that are visualized by TEM, bind Congo red and demonstrate apple-green birefringence in polarized light. Thus, we have obtained comprehensive evidence of the amyloid-like properties of the Toh1 protein. Taking into account that *toh1* null mutants are characterized with elevated sensitivity to a number of chemical agents which may be associated with a violation of the function of the cell wall [52,53] we can speculate that the Toh1 protein may be functional in yeast cells in a form of detergent-resistant amyloid-like aggregates. This is also in accordance with the data that different exposures violating the cell wall structure result in the elevating the expression of the *TOH1* gene [54–56]. The results of our work allow us to speculate also that Toh1 included in functional aggregates may be linked with the cell wall in two ways: 1) one part of protein molecules is covalently linked with the cell wall glycane scaffold by means of GPI anchor, and 2) another part of protein is linked with the GPI-anchored Toh1 molecules through hydrogen bonds, forming SDS-resistant amyloid-like aggregates (Figure 5). The second process is probably highly efficient and, as a result, the majority of the Toh1-YFP protein is revealed in high-molecular SDS-resistant aggregates.

The results of our work along with earlier data tell that a whole set of the yeast cell wall proteins can be represented by amyloid aggregates. At least 6 cell wall proteins of *S. cerevisiae*, Bgl2, Ygp1, Gas1, Flo1, Muc1, and Toh1, display amyloid-like properties in different assays [29,32,33]. Yeast cell wall is enriched with proteins prone to amyloid aggregation compared to most other cell components [57,58]. Such a broad representation of amyloid-like proteins in the cell wall may have biological significance, the detailed elucidation of which is the subject of further research. We can presume that amyloid-like aggregates in the cell wall can provide it with both additional strength and elasticity due to the physico-chemical properties of amyloids. It is possible that elongation and/or dissolution of amyloid fibrils resided in a cell wall may be controlled in response to different exposures. In addition, possible dual mechanism of incorporation of some amyloid-forming proteins into the cell wall mentioned above, both through covalent linkage with glycanes and through formation of amyloid-like aggegates of protein molecules not linked covalently with glycanes (Figure 5), may provide the cell wall structure with additive lability. As only small part of protein molecules in the amyloid-like aggegates may be linked covalently with glycan scaffold, the fibrils may be quickly erased from the cell surface by cutting relatively small number of covalent links, and replaced with the aggregates of another amyloid protein(s) when environmental conditions change or cell enters another stage of life cycle.

It is worth noting that Toh1, as well as some other amyloid-like proteins of the yeast cell wall (e.g., Gas1, Muc1), resemble Prp, well-known mammalian prion protein, in the sense that they are all GPI-anchored proteins with amyloid properties. This fact explores the possibility to implicate these yeast proteins as a model to study amyloidogenesis peculiarities associated with GPI-anchored amyloid-forming protein.

We used the chimeric Toh1(20–365)-YFP protein expression in the yeast strains containing [*PSI*^+^] and [*PIN*^+^] prions as a model for studying interactions between amyloids with dissimilar primary structures, particularly between Q/N-rich and non-Q/N-rich amyloids. As Toh1(20–365) fragment lacks the N-terminal and C-terminal peptides determining Toh1 transport to and incorporation into the cell wall and/or cell membrane, the Toh1(20–365)-YFP is presumed to be localized in cytoplasm. We have found that the Toh1(20–365)-YFP protein demonstrates surprisingly strong colocalization with amyloid aggregates of the Rnq1 and Sup35 prion domains fused with CFP in strains containing endogenous prions [*PSI^+^*] and [*PIN^+^*]. Moreover, the FRET efficiency values for the Toh1(20–365)-YFP/Rnq1C-CFP and Toh1(20–365)-YFP/Sup35NM-CFP pairs indicate the existence of a physical interaction between these aggregated proteins. The question of whether there is the interaction of the intact Toh1 protein with Rnq1 and/or Sup35 aggregates under physiological conditions requires more thorough research. Probably, such interaction may take place if some part of Toh1 protein is localized in the cytoplasm and/or in the cell membrane facing the inside of the cell. In this case presence of the prion aggregates of Rnq1 or Sup35 may to some extent interfere with the localization of the Toh1 in the aggregates at the cell wall. The possibility that not all Toh1 protein functions in the cell wall and cell membrane indirectly follows from the data on the increase in Toh1 production in response to replicative stress [59].

The observed interaction of the Toh1(20–365)-YFP protein with Rnq1C-CFP and Sup35NM-CFP, on the one hand, is an additional argument confirming the amyloid nature of Toh1 protein as different amyloids often tend to interact with each other. On the other hand, we present here one more example of the interaction between Q/N-rich and non Q/N-rich amyloid proteins that have no similarity in their primary structure. Thus, our data suggest that possibility of interaction between different amyloid proteins is determined not only by their sequence similarity but as well by similarity of their conformational folds.
