# Supplementary figures and images for: Amyloid properties of the yeast cell wall protein Toh1 and its interaction with prion proteins Rnq1 and Sup35
Source: Prion. 2018 Dec 27;13(1):21–32. doi: 10.1080/19336896.2018.1558763 (PMC6422396; doi:10.1080/19336896.2018.1558763)

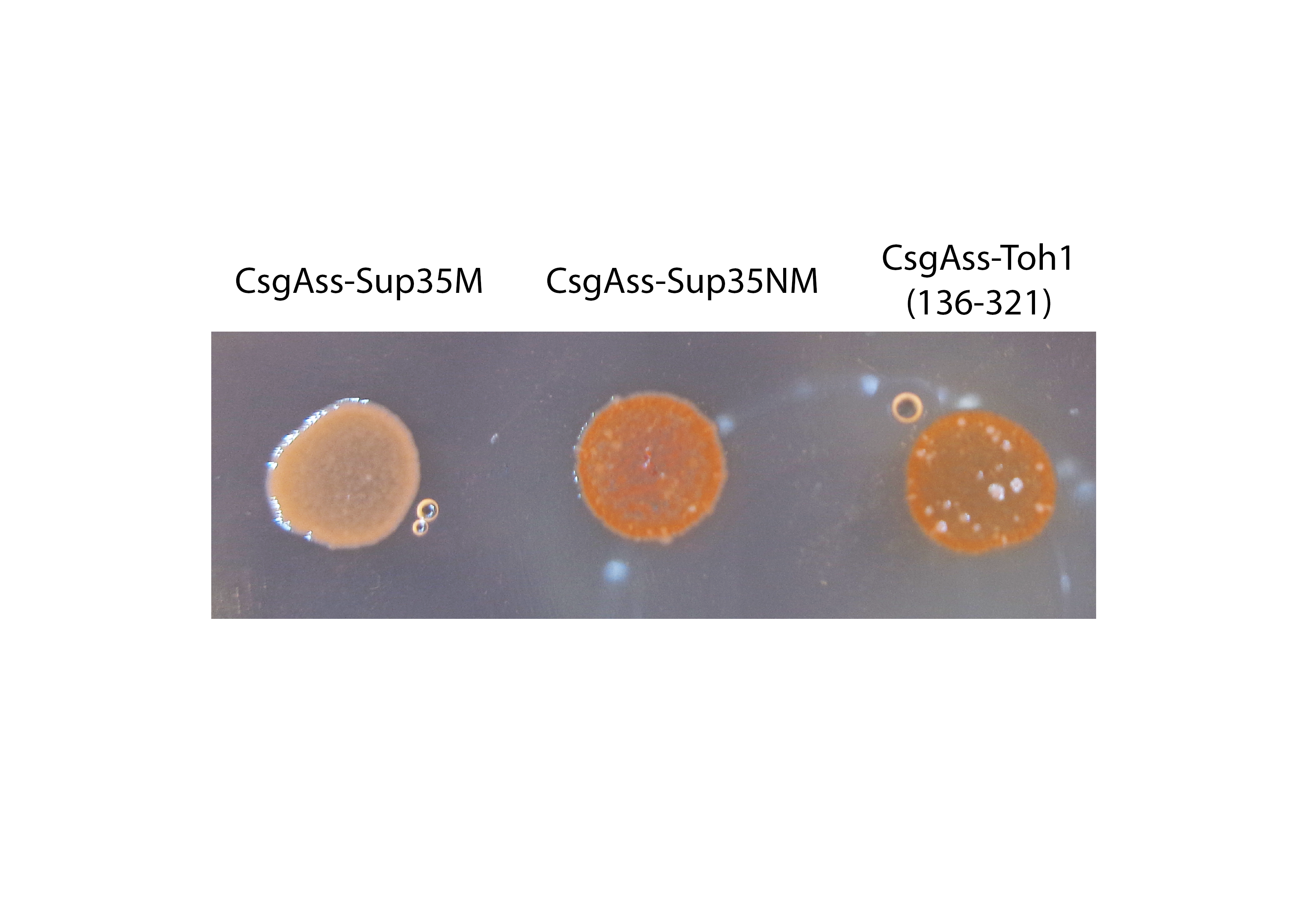

Supplement: Supplemental Material [file kprn-13-01-1558763-s001.zip › Supplementary information/fig S1.tif]

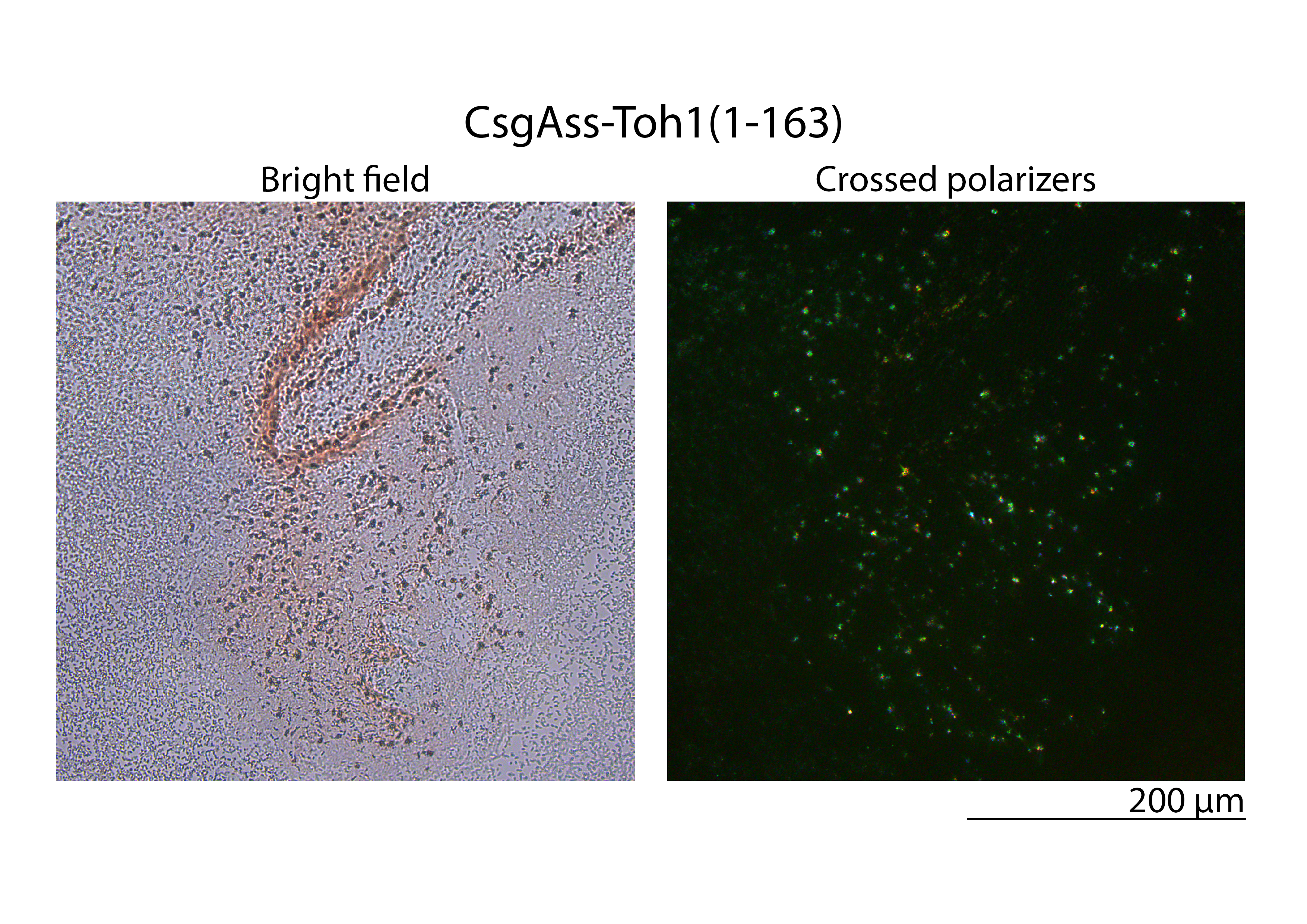

Supplement: Supplemental Material [file kprn-13-01-1558763-s001.zip › Supplementary information/fig S2.tif]

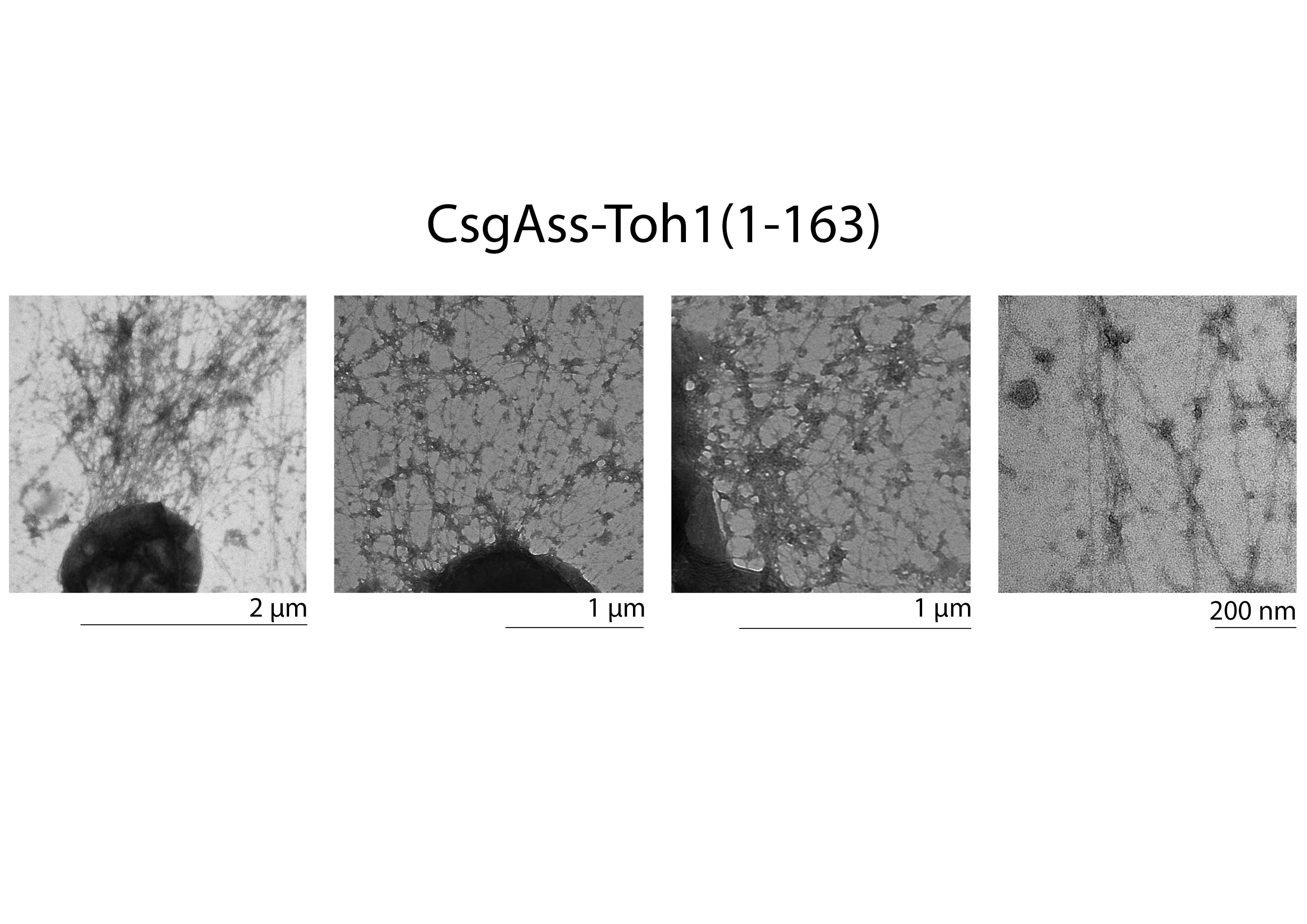

Supplement: Supplemental Material [file kprn-13-01-1558763-s001.zip › Supplementary information/fig. S3.tif]

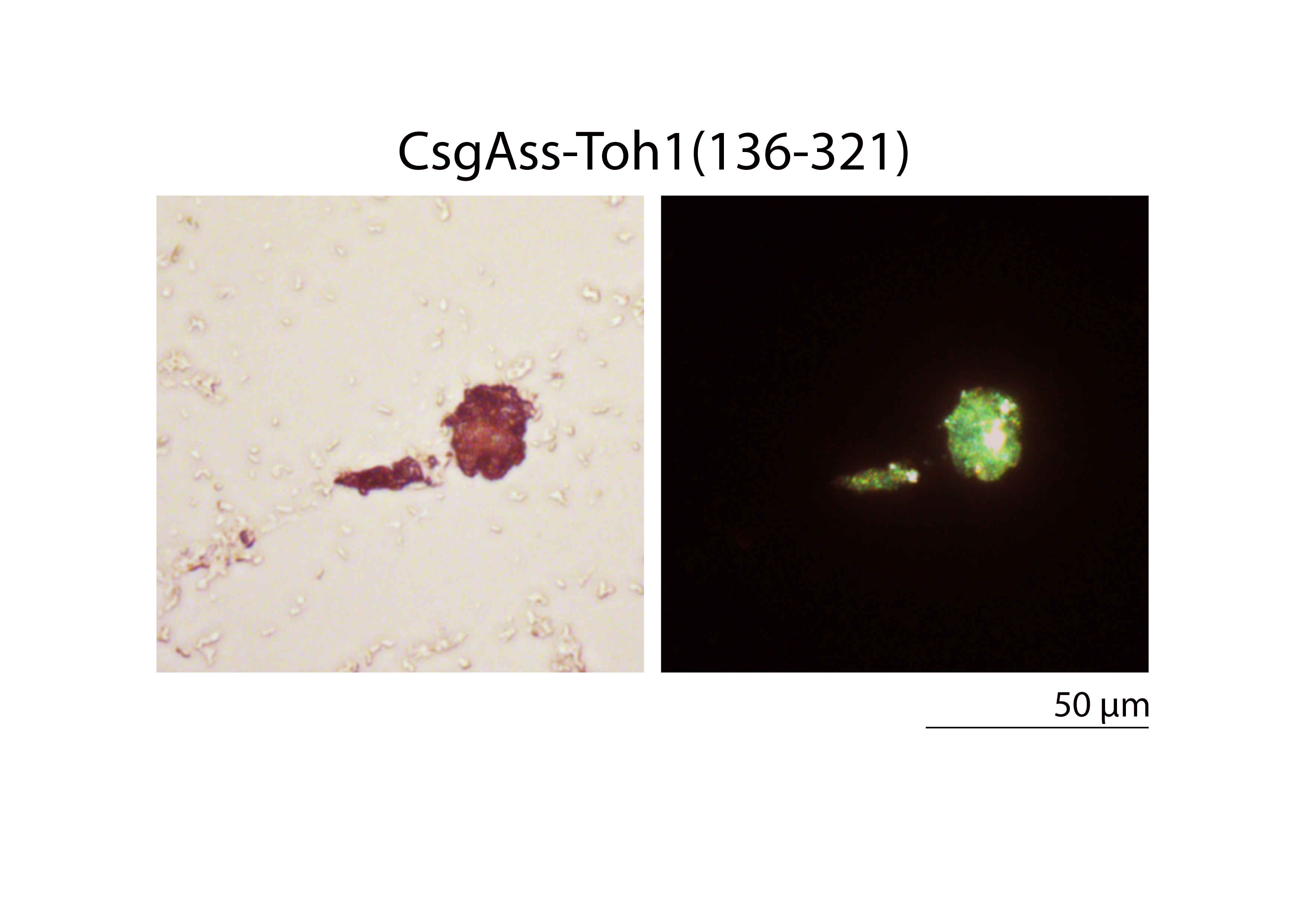

Supplement: Supplemental Material [file kprn-13-01-1558763-s001.zip › Supplementary information/fig. S4.tif]

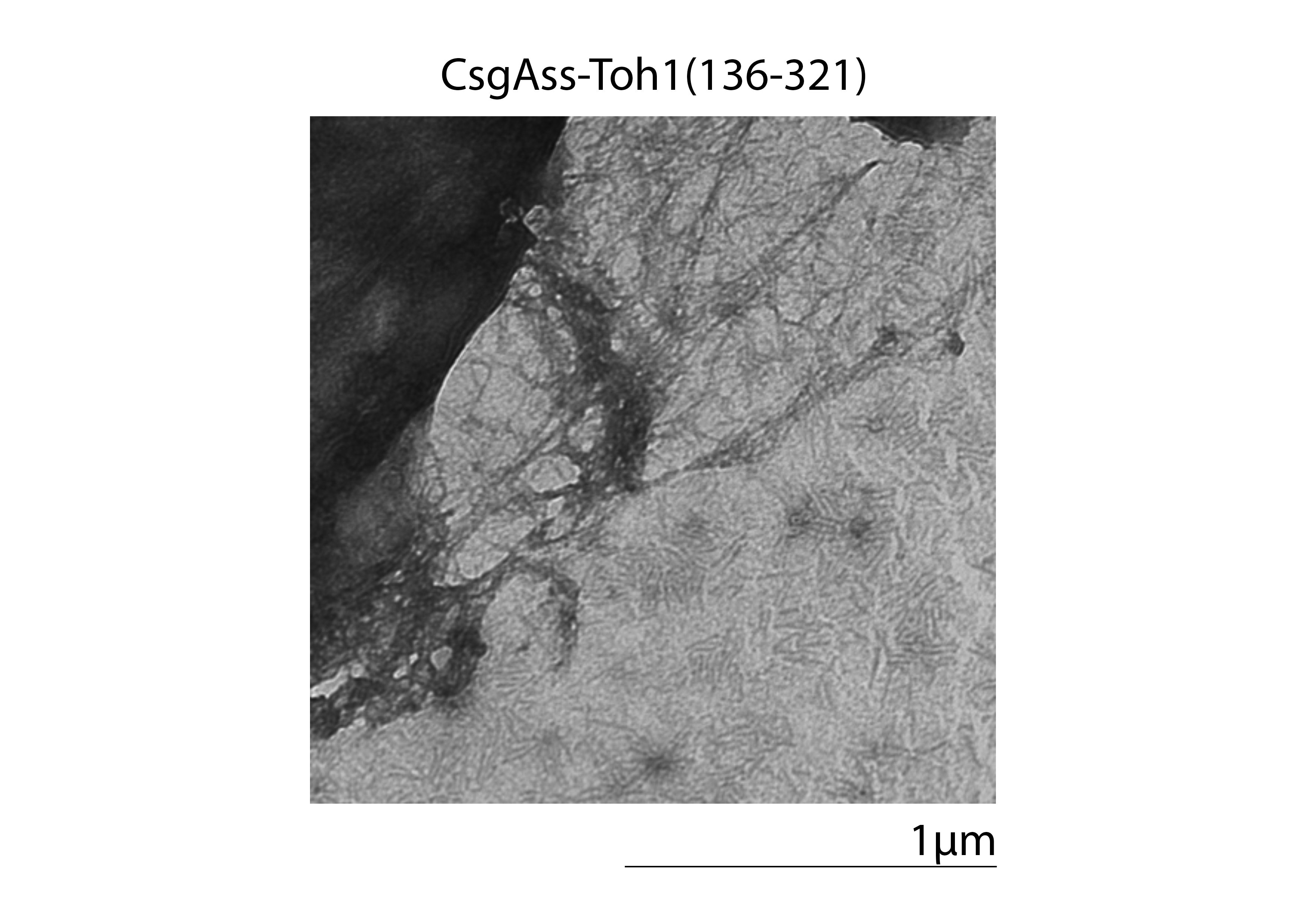

Supplement: Supplemental Material [file kprn-13-01-1558763-s001.zip › Supplementary information/fig. S5.tif]
