# Supplementary material for: Amyloid properties of the yeast cell wall protein Toh1 and its interaction with prion proteins Rnq1 and Sup35
Source: Prion. 2018 Dec 27;13(1):21–32. doi: 10.1080/19336896.2018.1558763 (PMC6422396; doi:10.1080/19336896.2018.1558763)
Supplement: Supplemental Material [file kprn-13-01-1558763-s001.zip › Supplementary information/Supplementary material.doc]

**Supplementary material**

**Figure legends**

**Fig. S1.**

Colony color assay of the cells when plated on agar containing CR showing CR binding for colonies expressing CsgAss-Toh1(136–321) and CsgAss-Sup35NM.

**Fig. S2.**

Micrographs of CsgAss-Toh1(1–163) scraped cell sample harvested from CR-containing agar. Extracellular material binds CR (left) and displays apple-green birefringence when viewed between crossed polarizers (right).

**Fig. S3.**

Electron micrographs of amyloid fibrils formed by secreted CsgAss-Toh1(1–163) protein. Scale bar, 200 nm to 2 mkm.

**Fig. S4.**

Micrographs of CsgAss-Toh1(136–321) scraped cell sample harvested from CR-containing agar. Extracellular material binds CR (left) and displays apple-green birefringence when viewed between crossed polarizers (right).

**Fig. S5.**

Electron micrograph of amyloid fibrils formed by secreted CsgAss-Toh1(136–321) protein. Scale bar, 1 mkm.
